# Genetic variation of wild and hatchery populations of the catla Indian major carp (*Catla catla* Hamilton 1822: Cypriniformes, Cyprinidae) revealed by RAPD markers

**DOI:** 10.1590/S1415-47572009005000013

**Published:** 2009-01-16

**Authors:** S. M. Zakiur Rahman, Mukhlesur Rahman Khan, Shahidul Islam, Samsul Alam

**Affiliations:** 1 Department of Biotechnology, Bangladesh Agricultural University, MymensinghBangladesh; 2 Department of Fisheries Biology and Genetics, Bangladesh Agricultural University, MymensinghBangladesh

**Keywords:** * Catla catla*, genetic variation, RAPD markers

## Abstract

Genetic variation is a key component for improving a stock through selective breeding programs. Randomly amplified polymorphic DNA (RAPD) markers were used to assess genetic variation in three wild population of the catla carp (*Catla catla* Hamilton 1822) in the Halda, Jamuna and Padma rivers and one hatchery population in Bangladesh. Five decamer random primers were used to amplify RAPD markers from 30 fish from each population. Thirty of the 55 scorable bands were polymorphic, indicating some degree of genetic variation in all the populations. The proportion of polymorphic loci and gene diversity values reflected a relatively higher level of genetic variation in the Halda population. Sixteen of the 30 polymorphic loci showed a significant (p < 0.05, p < 0.01, p < 0.001) departure from homogeneity and the *F*_ST_ values in the different populations indicated some degree of genetic differentiation in the population pairs. Estimated genetic distances between populations were directly correlated with geographical distances. The unweighted pair group method with averages (UPGMA) dendrogram showed two clusters, the Halda population forming one cluster and the other populations the second cluster. Genetic variation of *C. catla* is a useful trait for developing a good management strategy for maintaining genetic quality of the species.

The catla carp (*Catla catla* Hamilton 1822: Cypriniformes, Cyprinidae) is an Indian major carp and one of the major aquaculture species of Bangladesh, India, Myanmar (Burma) and Pakistan (Jhingran, 1968). In Bangladesh, *C. catla* is mostly found in the Gangetic and the Halda river systems in the hilly Chittagong region of eastern Bangladesh and is the second most popular indigenous carp species in Bangladesh due to its relatively good taste and high market price. Indian major carps, principally *C. catla, Cirrhinus mrigala* and *Labeo rohita*, accounted for about 5,09,995 tonne in Bangladesh in 2005-2006 (DoF, 2006). However, as a principal species, *C. catla* only contributed about 1,235,992 tonne to total world aquaculture production in 2005 (FAO, 2007).

The natural breeding of *C. catla* has become uncertain due to continuous degradation of habitat caused by environmental modification and manmade interventions affecting spawning and feeding migration, leading to decreased *C. catla* populations in all Bangladeshi rivers (Das and Barat, 1990) and a reduction in natural spawn production, with less than 1% of wild *C. catla* females spawning as compared with 80% in the early 1980s (DoF, 2003). However, river-derived fry have shown much better growth performance than hatchery produced fry (Shah and Biswas, 2004). Inbreeding is a common scenario in Bangladeshi hatcheries and hybrids are produced intentionally or unintentionally in the many carp hatcheries (Simonsen *et al.*, 2005), with the mass stocking of genetically inferior fry into open water bodies having the potential to cause feral gene introgression into the pure wild stocks. Thus *C. catla* may be prone to loss of genetic diversity and variability due to the extinction of genetically distinct wild populations as a result of the escape of hatchery-reared fish or the ranching of fry (Ponniah, 1997).

The identification of pure *C. catla* stock using molecular markers based on allozyme analysis, microsatellites, mitochondrial DNA (mtDNA), randomly amplified polymorphic DNA (RAPD), restriction fragment length polymorphisms (RFLP) and allied methodologies are useful for piscine gene pool conservation and enhancement of aquaculture production of fish species (Welsh and McClelland, 1990; Liu and Cordes, 2004). The RAPD technique involves amplification of target DNA by the polymerase chain reaction (PCR) using arbitrary primers and is useful for studying variation in species with low genetic variability when other techniques such as isozyme analysis and mtDNA control region sequencing fail to reveal differences between individuals (Bowditch *et al.*, 1994). Fundamental DNA marker studies on genetic variation in riverine and hatchery populations of *C. catla* in Bangladesh have been carried out using microsatellites (Alam and Islam, 2005) and RAPD markers (Islam *et al.*, 2005).

The variation detected in *C. catla* could help in formulating more effective strategies for managing this aquaculture species and also in evaluating the potential genetic effects induced by hatchery operations for selective breeding. The objective of the study described in the present paper was to assess genetic variation and relatedness in three wild riverine and one hatchery *C. catla* population using RAPD markers.

In July 2005 we collected 250 g samples of 4 day to 5 day old *C. catla* spawn from each of four different sources (Figure 1): the Halda river (22°25'0” N, 91°53'0” E, altitude 81000 m), the Padma river (30°54'0” N, 79°7'0”, altitude 3900 m) and the Jamuna river (31°30'0” N, 82°0'0” E, altitude 205000 m) and one hatchery on the Brahmaputra river (31°30'0” N, 82°0'0” E, altitude 6900 m) and transported to the Field Laboratory Complex of the Faculty of Fisheries, Bangladesh Agricultural University, Mymensingh. The spawns were reared for two months in four different rectangular ponds (9.5 m x 6.1 m x 0.8 m) and spawns were supplied with nursery feed (Catla Nursery Feed, Saudi-Bangla Fish Feed Bangladesh Ltd., Bangladesh) containing 30% protein for the first two weeks, followed by a common supplemental feed administered at 10% of body weight per day. After 60 days each pond contained about 180 fish.

We randomly sampled 30 unsexed juvenile fish (length = 7.18 cm ± 0.43 cm; mass = 43.68 g ± 5.27 g) from each pond (representing one distinct population) for RAPD marker analysis. Caudal fin tissue samples were clipped from each fish and preserved in 95% ethanol.

Genomic DNA was extracted from fin tissue following the standard method of Islam and Alam (2004). The quality of extracted DNAs was assessed by electrophoresis on 1% (w/v) agarose gel and the concentration determined using a Spectronic^®^ Genesys5 UV-spectrophotometer (Spectronic Instruments Inc., USA). We used sub-samples of three fish from each population to screen 27 random sequence decamer primers (GENEI Pvt. Ltd., Bangalore, India), 10 of which were selected on the basis of the intensity or resolution of bands without smearing, repeatability of markers and consistency within individual fish. Of the 10 pre-selected primers, seven, exhibiting the highest quality banding patterns, were used for analysis of all the samples.

Initial PCR amplifications were ran to test the effect of template DNA, dNTPs, Mg^++^, and Taq DNA polymerase concentrations and to determine the optimum annealing temperature essentially following the method described by Williams *et al.* (1990) with some modifications in annealing temperature. The PCR reactions were performed in an oil-free Mastercycler Gradient thermocycler (Eppendorf, Germany).

All distinct fragments were given identification numbers according to their size as measured by the DNAfrag software version 3.03 (Nash, 1991) and scored separately for each sample and primer on the basis of their presence (1) or absence (0). The scores obtained for all primers were then used to create a single data matrix for estimating the proportion of polymorphic loci, Nei's (1973) gene diversity (*H*), gene flow (*N*_m_) and homogeneity test at different loci using the POPGENE software version 1.31 (Yeh *et al.*, 1999). The Tools for Population Genetic Analyses (TFPGA) program version 1.3 (Miller, 1997) was applied for the estimation of population differentiation by the Reynolds coancestry coefficient (*F*_ST_ or θ) (Reynolds *et al.*, 1983) and the calculation of the 95% confidence interval (CI) from bootstrapping over loci using 1000 replications. Genetic distance was evaluated by several different methods contained in the Phylogeny Inference Package (PHYLIP) software version 3.67 (Felsenstein, 2007), the methods used being Cavalli-Sforza's chord measure (*D*_CH_) (Cavalli-Sforza and Edwards, 1967), Nei's genetic distance (*D*) (Nei, 1972) and Reynolds coancestry coefficient (θ). The same software was also used to construct unweighted pair-group method with averages (UPGMA) dendrograms (Sneath and Sokal, 1973) based on the estimated genetic distances. Significance tests for correlation between genetic and geographic distances were determined using Mantel tests (Mantel, 1967) implemented in the Mantel-Struct software version 1.0 (Miller, 1999). Band-sharing based similarity indices between individual fish within a population (*S*_i_) and between populations (*S*_ij_) were calculated according to the formula given by Lynch (1991).

Of the 27 decamer primer sets initially tested, seven primers (OPA01, OPA06, OPB02, OPAB05, OPE09, OPW16 and OPU20) yielded more numerous bands with high intensity and minimal smearing, although two (OPAB05 and OPU20) produced monomorphic bands and were excluded from the analysis. The five remaining primers generated 55 distinct bands, 30 of which were polymorphic. The frequency of the most common allele was 0.95 or less.

The overall proportion of polymorphic loci (*P*) and Nei gene diversity (*H*) (Nei, 1973) across all the primers in the studied *C. catla* populations were 54.55% and 0.208 respectively. Among the wild populations, the proportion of polymorphic loci gene diversity values were found to be the highest (p = 54.55%, *H* = 0.232) in the Halda river population, followed by the Padma (p = 49.09%, *H* = 0.198) and Jamuna river (p = 45.45%, *H* = 0.172) populations. However, the lowest percentage of polymorphic loci (p = 41.82%) and less gene diversity (*H* = 0.174) was found in the hatchery population.

Within population similarity was marginally higher (mean *S*_i_ = 87.86%) than between population similarity (mean *S*_ij_ = 87.07%). The *S*_i_ value was found to be highest in the Jamuna population (89.15%) followed by the hatchery (88.15%), the Padma (87.44%) and the Halda (86.72%) populations. On the other hand, the *S*_ij_ value for the Jamuna-Padma population pair was the highest (88.74%) compared with all the other between population comparisons. The highest gene flow (*N*_m_) was between the Padma and hatchery populations (*N*_m_ = 18.16), while the lowest was for the Halda-hatchery population pair (*N*_m_ = 6.34). Among the wild populations, the highest *N*_m_ value (17.17) was between the Padma and Jamuna river populations. No significant population differentiation (*F*_ST_) was found in the studied populations at the 95% confidence interval (CI) (Table 1). Of the 30 RAPD markers, 16 showed a significant departure from homogeneity in the population pairs.

Genetic distance, as calculated from combined data sets for five primers, ranged from *D*_*CH*_ = 0.043 to 0.145, *D* = 0.052 to 0.136 and θ = 0.013 to 0.040 (Table 2). All the genetic distance methods showed that the Jamuna population was closest to the Padma population. A direct correlation was tested between genetic distance and geographic distance of pairwise populations using the Mantel test (Table 2) and significant correlations were found between the Halda-Padma (p < 0.05), the Halda-Jamuna (p < 0.01), the Halda-hatchery and the Jamuna-hatchery (p < 0.001) populations, whereas no significant correlations were observed for the Jamuna-Padma and the Padma-hatchery population pairs.

A representative UPGMA dendrogram based on Nei's genetic distance (Nei, 1972) is shown in Figure 2. The dendrogram segregated the four *C. catla* populations into two distinct clusters, the Halda population being alone in one cluster while the Jamuna, Padma and hatchery populations belonged to the other cluster. The second cluster was further separated into two subgroups, the Jamuna population in one sub-group and the Padma and the hatchery populations in the other sub-group.

Islam *et al.* (2005) have previously studied the genetic variation of 30 *C. catla* from three wild populations in the Halda, Padma and Jamuna rivers in Bangladesh using four decamer random primers (OPB03, OPB08, OPB09 and OPB15) and reported 24 RAPD polymorphic loci and some degree of genetic variation. In our present study, to obtain a more precise knowledge of the genetic variation in *C. catla*, we included one hatchery population together with three river populations and screened a total of 120 fish and used five different random primers (OPA01, OPA06, OPB02, OPE09 and OPW16). Our study revealed 30 polymorphic loci, indicating a high level of polymorphism in the four *C. catla* populations studied and supporting the suitability of RAPD markers for effectively discriminating different populations. Moreover, our present study compared the genetic variation between hatchery and river populations, which might assist in formulating good management practices for increased aquaculture production.

We found that 54.55% of the loci in our study were polymorphic as compared to the 75% reported by Islam *et al.* (2005), this difference possibly being due to the relatively large sample sizes and the inclusion of a hatchery population in our study. However, our results are not atypical since in a study of four Indian major carps (*Catla catla*, *Cirrhinus mrigala*, *Labeo rohita* and *Labeo calbasu*) an average of 45% of loci were polymorphic (Barman *et al.*, 2003) and a study of 140 fish in four different *L. rohita* populations using five RAPD primers also found that 46.5% of loci were polymorphic (Islam and Alam, 2004). Our study certainly showed a relatively high level of genetic polymorphism in terms of the proportion of polymorphic loci, intra-population similarity indices and Nei's gene diversity (*H*) in the wild populations, especially the Halda and Padma populations, compared to the hatchery population. The Halda is a fresh water tidal river which is one of the principal spawning grounds of Indian major carps (Islam and Alam, 2004). Similarly, the Padma and the Jamuna are two major natural sources of Indian major carps in Bangladesh. These three wild stocks were considered as large random mating populations having an infinite number of effective individuals (*N*_e_) that possibly resulted in higher genetic variation. Lower levels of genetic variation in the hatchery population might be due to, among other factors, inbreeding, bottlenecking or genetic drift. To keep the maintenance costs to a minimum farmers maintain a smaller number of broods in most hatcheries and thus induce breeding between related fish, which means that the genetic variation of hatchery reared fish may be reduced over a period of captive management in the hatcheries. Similar findings to ours were reported in a population genetic analysis of *L. rohita* (Eknath and Doyle, 1990; Islam and Alam, 2004).

In our study, there was no significant population differentiation (*F*_ST_) in each population pair, with different *F*_ST_ values and significant deviations from homogeneity at a number of loci in the different populations indicating some degree of population differentiation in the different population pairs (Table 1). This contrasts with the work of Islam and Alam (2004), who detected significant differentiation at only 4 out of 43 loci in four *L.rohita* populations.

In spite of presenting some differences in genetic distances, the three methods for estimating genetic distances (*D, D*_*CH*_ and θ) produced UPGMA dendrograms with similar clustering patterns. Mantel tests detected correlations between genetic and geographical distances for some population pairs, with large genetic distances being found between distantly located populations. The Halda, an isolated river in south-eastern Bangladesh, is far from the other three populations, resulting in larger genetic distances between the Halda and any one of the other three populations. Contrastingly, the close relationship between the Padma and Jamuna population pair, as shown by the small genetic distance between this pair, may have been due to the connection between these two rivers (Figure 1) and the fact that these rivers may merge during rainy season flooding when there is the possibility of intermixing of fish from these two rivers, possibly leading to high levels of gene flow and inter-population similarities between these two riverine populations. The lowest genetic distance was between the Padma river and the hatchery population pair, possibly indicating that the hatchery owner collected their brood fish from the Padma river. A similar population cluster has also been reported for *L. rohita* (Islam and Alam, 2004), although in this case genetic distance values were inversely related to inter-population genetic similarities and gene flows.

Our study shows that wild stocks of *C. catla* represent a diversified genetic resource and indicates that *in situ* management practices, such as preventing the wanton capture of fish and creating sanctuaries for protecting small stocks such as those in the Halda river, can help maintain and conserve the present diverse gene pool. Hatchery owners are accustomed to operating negative selection and polygynous breeding systems in which some males mate with many females year after year (Islam *et al.*, 2007), resulting in genetic deterioration that subsequently cause a negative impact on aquaculture production. Based on our present findings, hatchery owners can collect their brood fish or replace their existing breeding populations with genetically diverse fish from stocks like those in the Halda and the Jamuna rivers and increase their effective breeding populations and thus improve the aquaculture production. However, the strict implementations of correct management practices are essential to maintaining the genetic diversity of the natural stocks. A further important point is that breeding between two genetically discreet or distant populations may have a positive impact on aquaculture production.

In spite of still being used in assessing population structure, RAPD markers have many shortcomings, such as difficulty in demonstrating Mendelian inheritance of the bands (which are not true genetic loci), the inability to distinguish between homozygotes and heterozygotes, the presence of paralogous PCR products and the low reproducibility all limit the application of RAPD marker in some cases. To produce more precise data on the genetic structure of *C. catla* additional studies should be carried out using methods such as Amplified Fragment Length Polymorphism (AFLP) and simple sequence repeat (SSR) markers to better characterize populations of *C. catla* and other fish.

**Figure 1:**
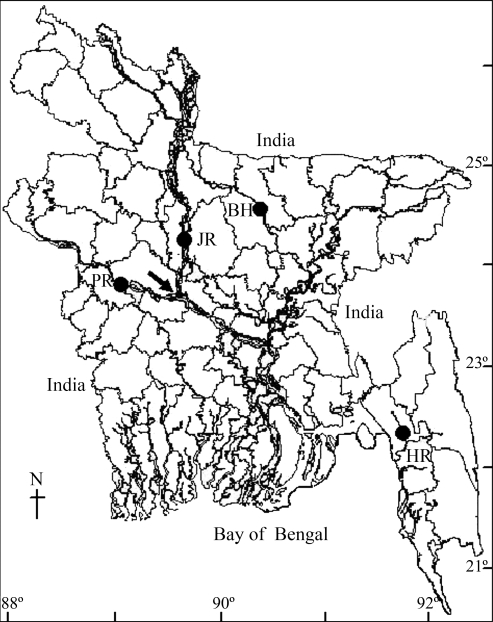
Map of Bangladesh showing the collection sites of catla (*Catla catla*) samples. The populations are referred as 1. Halda River (HR), 2. Jamuna River (JR), 3. Padma River (PR) and 4. Brahmaputra hatchery (BH). Arrow indicates the joining point between the Padma and the Jamuna rivers.

**Figure 2:**
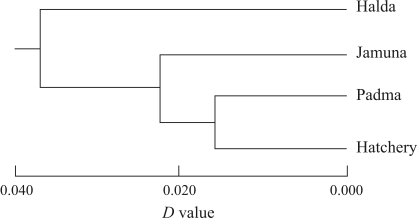
Unweighted pair-group method with averages (UPGMA) dendrograms based on Nei's *D* value (Nei, 1972) original measures of genetic distance, summarizing the data on differentiation between *Catla catla* populations according to RAPD analysis.

## Figures and Tables

**Table 1:** Population differentiation (θ or *F*_ST_) based on a square root transformation of the frequencies of the recessive null allele genotype by the 95% confidence interval (CI) (below diagonal).

Populations	Halda	Jamuna	Padma
Jamuna	0.082 ± 0.026 (0.035-0.138)		
Padma	0.052 ± 0.023 (0.012-0.098)	0.054 ± 0.020 (0.023-0.094)	
Hatchery	0.118 ± 0.032 (0.063-0.185)	0.079 ± 0.031 (0.025-0.145)	0.033 ± 0.015 (0.008-0.066)

**Table 2:** Three different measures of genetic distance between *Catla catla* populations. Significance levels (p-values) for the correlation between genetic distances and geographical distances using the Mantel test are given in the last column. Statistically significant values are marked with asterisks.

Population pairs	Cavalli-Sforza chord measure (*D*_CH_)	Nei's genetic distance (*D*)	Reynolds genetic distance (θ)	p-values from pairwise comparisons
Halda - Jamuna	0.100	0.028	0.100	0.0030**
Halda - Padma	0.066	0.021	0.071	0.0440*
Halda - Hatchery	0.145	0.040	0.136	0.0010***
Jamuna - Padma	0.043	0.013	0.055	0.2527
Jamuna - Hatchery	0.070	0.023	0.098	0.0010***
Padma - Hatchery	0.053	0.013	0.052	0.1928
Overall populations	0.043-0.145	0.013-0.040	0.052-0.136	-

*p < 0.05, ** p < 0.01, *** p < 0.001.
